# Novel Comparative Study for the Detection of COVID-19 Using CT Scan and Chest X-ray Images

**DOI:** 10.3390/ijerph20021268

**Published:** 2023-01-10

**Authors:** Ahatsham Hayat, Preety Baglat, Fábio Mendonça, Sheikh Shanawaz Mostafa, Fernando Morgado-Dias

**Affiliations:** 1 University of Madeira, 9000-082 Funchal, Portugal; 2 Interactive Technologies Institute (ITI/LARSyS and ARDITI), 9020-105 Funchal, Portugal

**Keywords:** COVID-19, CT scan, chest X-ray, machine learning, deep learning

## Abstract

The number of coronavirus disease (COVID-19) cases is constantly rising as the pandemic continues, with new variants constantly emerging. Therefore, to prevent the virus from spreading, coronavirus cases must be diagnosed as soon as possible. The COVID-19 pandemic has had a devastating impact on people’s health and the economy worldwide. For COVID-19 detection, reverse transcription-polymerase chain reaction testing is the benchmark. However, this test takes a long time and necessitates a lot of laboratory resources. A new trend is emerging to address these limitations regarding the use of machine learning and deep learning techniques for automatic analysis, as these can attain high diagnosis results, especially by using medical imaging techniques. However, a key question arises whether a chest computed tomography scan or chest X-ray can be used for COVID-19 detection. A total of 17,599 images were examined in this work to develop the models used to classify the occurrence of COVID-19 infection, while four different classifiers were studied. These are the convolutional neural network (proposed architecture (named, SCovNet) and Resnet18), support vector machine, and logistic regression. Out of all four models, the proposed SCoVNet architecture reached the best performance with an accuracy of almost 99% and 98% on chest computed tomography scan images and chest X-ray images, respectively.

## 1. Introduction

The coronavirus disease (COVID-19) has caused a pandemic since it first surfaced in late 2019 in Wuhan, Hubei Province, China. It has had a significant impact worldwide, affecting many elements of modern life. After causing a severe pneumonia outbreak in China, the International Committee on Taxonomy of Viruses named the novel coronavirus Severe Acute Respiratory Syndrome Coronavirus 2 (SARS-CoV-2) [1,2,3]. According to the World Health Organization, COVID-19 can be passed from person to person. Due to its rapid spread nature, on 30 January 2020, the World Health Organization (WHO) declared the COVID-19 virus a Public Health Emergency of International Concern (PHEIC) [4]. Given the magnitude and potential repercussions of COVID-19, as well as the fact that infected patients may spread swiftly if not isolated immediately, sensitive and accessible testing procedures are crucial. The most frequent presenting symptoms that may be seen in COVID-19 patients, such as dry cough, shortness of breath, and sore throat, etc. can be observed using medical imaging such as chest X-rays (CXR), and chest computed tomography (CT) scans [5]. In order to minimize the virus’s transmission rate, curfews and lockdowns have been implemented by governments all around the world, restricting people’s mobility, products, and services, and asking for “work from home”. Despite considerable government actions such as lockdowns and the enforced use of face masks in public places, it was not possible to stop the spread of COVID-19 throughout the world [6]. Furthermore, it is becoming even more difficult to stop the spread as new variants of COVID-19 are emerging, and they spread very quickly. So, it is vitally important to find some other methods for COVID-19 detection that are reliable and fast.

COVID-19 is confirmed by a nucleic acid amplification test of the respiratory tract or blood samples using reverse transcription-polymerase chain reaction (RT-PCR) [7,8]. However, its sensitivity is restricted, and the result is heavily operator dependent. More significantly, it is extremely time consuming, which is a major problem as new variants of COVID-19 spread really fast. For this reason, it is imperative to find alternatives to the RT-PCR test. Automatic analysis of medical images, such as CXR and CT scans, can be used as an alternative, as the examination takes less time and is highly stable compared to the RT-PCR test. Even images are more reliable and less dependent on operators [9]. Furthermore, various studies show that COVID-19 has a significant and harmful impact on the lungs, even in the initial stages of the disease, which can be easily detected by a CT scan. Likewise, several investigations have shown that, in addition to clinical symptoms, both blood and biochemical testing and lung CT imaging are useful diagnostic tools for determining the condition [9]. Although some studies have also shown that CXR can be used in conjunction with the RT-PCR test to assess problems [10], the widespread use of computer-aided diagnostic systems that use medical images, such as CT scans and CXR pictures, is currently impractical due to the lack of automatic scoring algorithms that can aid in early COVID-19 diagnoses [11].

**Figure 1:**
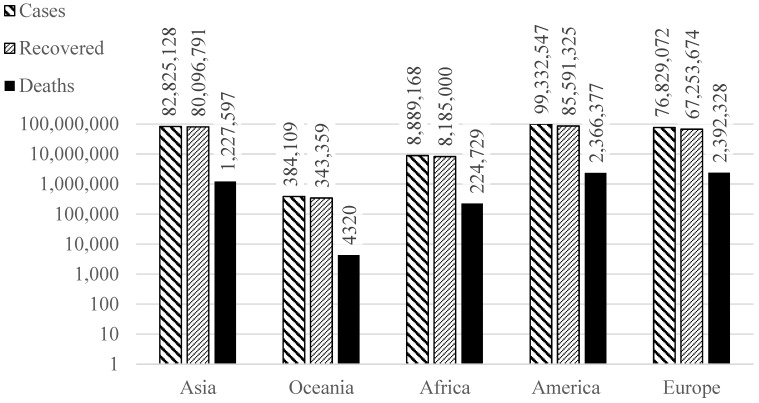
Stats of COVID-19 worldwide until the ninth of December 2021.

Considering the COVID-19 pandemic, it is critical to have automatic diagnosis tools, such as an artificial intelligence (AI)-based healthcare system, to expedite the search for positive cases in the population [12,13,14]. Numerous works have demonstrated that AI has been widely used to predict COVID-19 by utilizing conventional machine learning and deep learning algorithms. Different AI approaches have been developed in recent years, dramatically altering the landscape of numerous academic domains. AI-based solutions in healthcare are already challenging long-held beliefs within the healthcare sector. Specifically, it was observed that deep learning algorithms on medical pictures such as CXR, CT scan, and brain MRI could yield excellent results with high accuracy [15,16,17]. Deep learning, considered a significant achievement in the area of artificial intelligence during the previous decade, has immense potential for extracting microscopic information by fundamental units. When scanning various human organs, CXR and CT scan devices are well known for offering cost-effective and rapid conclusions. In hospitals, these images are often interpreted manually by a professional. Developing models based on artificial intelligence algorithms with images collected from these patients can significantly help specialists easily recognize COVID-19 patients. It can also benefit underdeveloped nations where CXR and CT scan facilities are accessible, but access to an expert is problematic [18]. Figure 1 depicts the worldwide numbers of COVID-19, where it is notorious that it affects all continents, leading to a considerable number of deaths, even in the most developed countries, thus strongly advocating the need for automatic detection tools.

Image classification plays a significant role in automatic medical image processing, especially in the case of CXR or CT scan images. During these processes, one or more images are taken as an input sample, and pre-processing step, such as resizing, scaling, etc., is performed on those images. In the second phase, conventional machine learning and deep learning models will be trained on the dataset. In the last phase, the model will automatically classify between the different output classes.

A literature review observed that although various studies have performed CT scans and CXR images for COVID-19 detection, due to several limitations, such as a limited number of available datasets and poor image quality, it is questionable if they are providing the best output model. Hence, it is not clear which imaging method is more useful for COVID-19 disease detection. The main objective of this study is to carry out an analysis to assess which image method is better for different scenarios. For this purpose, two datasets, one composed of CT scan images and the other composed of CXR images, were fed to various conventional machine learning and deep learning models to check for the performance of each dataset.

## 2. Literature Review

To assess the state-of-the-art methods for COVID-19 detection using image-based classification, a literature review was performed. A total number of 56 relevant papers were found. Out of these searches, 44 irrelevant papers were excluded through screening abstracts and titles. A total of 12 articles were included in this study. These included papers that presented an application of machine and deep learning approaches using CT and CXR images to categorize the subjects as COVID-19 positive or negative. In this section, the identified articles which are using AI techniques for the detection of COVID-19 are discussed. 

Jiao et al. [19] employed a deep neural network and medical data to predict the binary outcomes of COVID-19 disease and severity using CXR images. The model achieved a lower accuracy on the external testing dataset compared to the internal testing dataset, which could be caused by different heterogeneous datasets and image acquisition procedures between different hospitals. AI-Waisy et al. [20] also used various machine learning and deep learning strategies for the detection of the COVID-19 virus using CXR images, achieving an accuracy of 99.99%. Various deep learning-based feature extraction frameworks were applied to obtain the most accurate models for COVID-19 detection using CXR and CT images. 

Using the DenseNet121 and ResNet50 feature extraction-based frameworks, Kassani et al. [21] attained a very high accuracy when validated on publicly available COVID-19 datasets (CT and CXR scans). However, the main limitation of this study is the small size of the used dataset (137 positives and 137 negative cases), which can lead to generalization problems in models. Alruwaili et al. [22] studied the Inception ResNet V2 model for the classification and detection of COVID-19, proposing to classify normal, COVID-19, and pneumonia patients. Similarly to Kassani et al. [21], the model reached a high accuracy of 99.83%. However, the number of COVID-19 patient samples was very small. Hemdan et al. [23] used Inception V3, MobileNetV2, VGG19, DenseNet201, Inception-RestNetV2, and Xception models for the classification and detection of COVID-19. The model was able to achieve good performance but in a very small dataset totaling 50 cases (25 normal patients and 25 COVID-19 patients).

Kavitha et al. [24] proposed a ResNet-100 Convolutional Neural Network (CNN) model with logistic regression to detect COVID-19 and normal cases, and the model achieved a good performance using CT scan images with COVID-19 positive cases. The fast nCOVnet model was used in Panwar et al. [25] study for COVID-19 detection based on CXR images. The study achieved an accuracy higher than 97% in 5 s as compared to the RT-PCR test, which takes hours to detect positive and negative COVID-19 patients. Nguyen et al. [26] proposed a deep learning tool for detecting COVID-19 using 3D CT scan images. The average time taken for COVID-19 detection for each model is 0.53 s, which makes the model useful for real-time application. They have used multiple datasets in their study and used nine different deep learning models with the combination of dataset.

Using CoroNet, a deep learning model pre-trained on the ImageNet dataset and based on Xception architecture, trained on CXR images, Khan A. I. et al. [27] achieved an accuracy lower than the other examined works. It is likely that such is due to the small number of samples in the dataset and the lack of more pre-processing steps. Bukhari et al. [28] reported that the ResNet50 model provided high accuracy, although it takes a long execution time relative to the other models due to the dense structure of internal segments. However, the dataset used by Bukhari et al. is small compared to other work. 

Rehman A. et al. [29] presented a systematic review of COVID-19 detection based on different machine learning and deep learning techniques. They found that only 40 studies are using machine learning and deep learning approaches, out of which the RT-PCR-based model with SVM gives the lowest accuracy of 80%, and the CXR-based model with deep learning gives the best accuracy of 99.7%. Furthermore, they have found some open issues which need more research in the future. 

Gauda W. et al. [30] proposed two deep learning approaches based on ResNet50 architecture for COVID-19 detection on CXR images. They have used data augmentation, enhancing, normalization, and resizing on pre-processing stage. The proposed architecture outperforms some of the traditional deep learning architectures such as VGG and DenseNet, with an accuracy of 99.63%.

The literature concluded that few studies had been conducted using conventional machine learning and deep learning approaches to confirm which image technique (CT scan or CXR) should be adopted for COVID-19 detection. This work addresses both identified gaps by examining several machine learning and deep learning models with a dataset significantly larger than the state-of-the-art works.

## 3. Methodology

**Figure 2:**
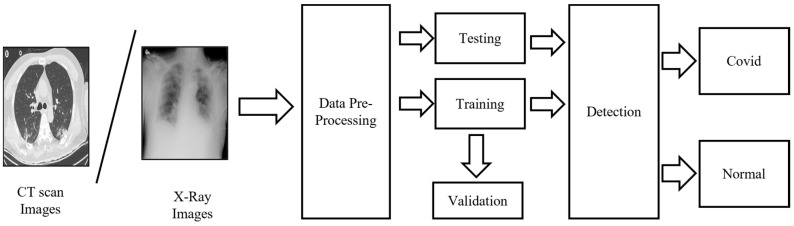
Steps for COVID-19 detection using image processing.

The proposed employed methodology uses CXR and CT scan data. Figure 2 illustrates the steps employed for the developed COVID-19 detection model. All code employed in this work was developed in Python, using scikit-learn and the TensorFlow library.

### 3.1. Dataset Description

**Figure 3:**
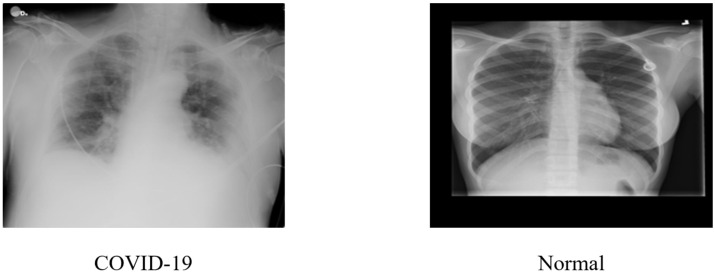
CXR images of the COVID-19 and healthy patient.

In this study, a comprehensive COVID-19 CXR and CT imaging collection were employed for COVID-19 identification [31]. It is an open-source dataset, available at Mendeley Data, composed of 17,599 images of COVID-19 and healthy cases, for both chest CT scans and CXR. The dataset contains 9544 CXR images, of which 5500 are of healthy persons, and 4044 are of COVID-19 patients. The CXR images presented in Figure 3 are examples of COVID-19 and healthy or normal cases.

**Figure 4:**
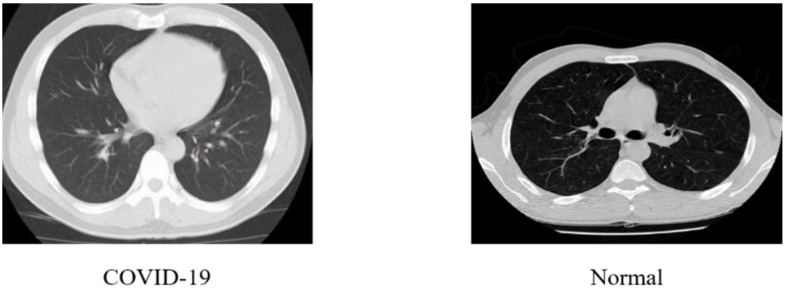
CT scan images of COVID-19 and healthy patients.

The second part of the dataset is composed of 8055 CT scan images which contain 2628 images of healthy persons and 5427 images of COVID-19 patients. The dataset was further split into three sets of data (training, testing, and validation), with a split of 85% for the training, 15% for the testing, and 15% for the validation purpose. Figure 4 shows examples of CT images of COVID-19 and healthy cases. 

### 3.2. Data Pre-Processing

The images are inconsistent since the dataset was obtained from numerous sources using different equipment and characteristics. Hence, pre-processing must be performed before giving to the model for processing. However, to increase the generalization ability of the proposed algorithm, this research avoids extensive pre-processing procedures. Therefore, two pre-processing steps were used to optimize the training process:

- Resizing: The CT scan and CXR dataset images vary in dimension and resolution. Therefore, in order to obtain a consistent dimension image, all of the images are resized to 224 × 224 pixels size.
- Image Normalization: For the consistent intensity of all the images, min–max normalization was used for intensity normalization. The intensity value of all images, in the range [0, 255], was normalized to the intensity range of [0, 1] by

(1) $$x_{N}=\frac{x-x_{min}}{x_{max}-x_{min}}$$

where *x* is the original value of pixels, while $x_{min}$ and $x_{max}$ are the minimum and maximum intensity values, respectively. This process will remove the bias from the features to help the model produce results without bias.

### 3.3. Classification

A total of four machine learning-based classifiers were examined in this work, varying from the conventional machine learning such as logistic regression and support vector machine to the deep learning models such as proposed Simple COVID-19 Neural Network (SCovNet) and pre-trained Resnet18 models. In the case of conventional machine learning, the size of the input image is 224 × 224 × 1. The goal is to allow a comparison between the methods and determine which is better for the intended analysis, taking into consideration the complexity of each model.

#### 3.3.1. Logistic Regression

The logistic regression model is a standard classifier for binary problems [32]. For this purpose, a function, *f*(*x*), composed of a linear combination of *r* estimators, *a*, was employed. This function is defined as

(2) $$f\left(x\right)=a_{0}+a_{1}x_{1}+\ldots +\;a_{r}x_{r}$$

With respect to *f*(*x*), the model will predict the record as COVID-19 if,

(3) $$a_{0}+a_{1}x_{1}+\ldots +a_{r}x_{r}\geq 0$$

The sigmoid function, *S*(*x*), was used as an activation function to map all the attributes with their respective classes. The sigmoid equation is given by

*S*(*x*) = 1/(1 + exp(−*f*(*x*)) (4)

According to Cedric et al. [33], logistic regression performed better when compared with other models such as random forest, decision tree, etc. A grid search methodology was then employed to optimize the tuning parameters of the model, and 0.5 was taken as the threshold value between COVID-19 and normal patient class. The examined parameters used in our study, set for the logistic regression, are penalty, random state, CV, and no. of iterations, and the selected values are L2, 42, 10, and 1000, respectively.

#### 3.3.2. Support Vector Machine

Support Vector Machine (SVM) was selected among the conventional machine learning algorithms previously employed for detecting COVID-19, as it can give more accuracy in general without the need for past knowledge [34]. In general, SVM performs two main tasks. First, used the kernel to map low-dimensional features to high-dimensional space, employing a linear classifier in the new space for the separation. Second, it tried to separate the data with an optimum hyperplane. Initially, an input image with the size of 224 × 224 × 1 will be fed to an input array. Binary SVM was used in this study for classification purposes, and the GridserachCV method was used to find out the best parameters for SVM. The GridseachCV method runs through all the parameters and gives the best combination for training the model. The Radial Basis Function (RBF) was used as a kernel function with the cost parameter (*C*) as 1 and the inverse of the standard deviation of the RBF kernel gamma (*γ*) as 0.1. The linear separation of COVID-19 and normal cases was achieved by Equation (5).

(5) $$K\left(x1,x2\right)=\exp{\left(-\gamma \left|\left|x1-x2\right|\right|\right)}^{2}$$

where *x*1 and *x*2 are two data points. ||*x*1 − *x*2|| is the Euclidean distance between *x*1 and *x*2.

#### 3.3.3. Convolutional Neural Network

**Figure 5:**
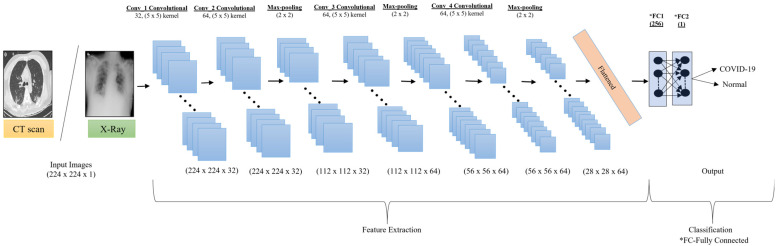
Proposed SCovNet architecture for COVID-19 detection.

The proposed SCovNet model was used to automatically identify the most meaningful features from the CT scan and CXR images and then perform the classification of the COVID-19 and healthy patients, as shown in Figure 5. The proposed SCovNet model contains 14 layers, out of which 4 perform the convolution operation (Conv2D layers), 3 are sub-sampling layers (performing the maximum pooling operation), 4 dropout layers avoid the problem of overfitting, 2 are fully connected layers, and 1 is a flattened layer. The input shape of the model is (224, 224, 1), and in all Conv2D layers, 5 × 5 kernel size was used. The filter size was 32 in the first Conv2D layer, while for the second, third, and fourth layers, it was 64. The last two fully connected (FC) layers include 256 neurons in the first (FC1) and 1 neuron in the second (FC2). To avoid overfitting, the max-pooling layer with a 2 × 2 pooling size and 0.5 dropout was applied after each Conv2D layer except the first Conv2D layer. Because the final pooling and convolutional layer produce a three-dimensional matrix as an output, the output was flattened using a flattening layer, converting it to a vector that will become the input of the first FC1 layer that uses Rectified Linear Unit (ReLU) as an activation function. The second and final FC2 layer uses the sigmoid activation function for the classification of COVID-19 patients. The total number of parameters is 13,104,193, and all of them are trainable parameters. The input dimension of the proposed SCovNet was 224 × 224 × 1, with a learning rate of 0.001 for training the model.

#### 3.3.4. ResNet18

**Figure 6:**
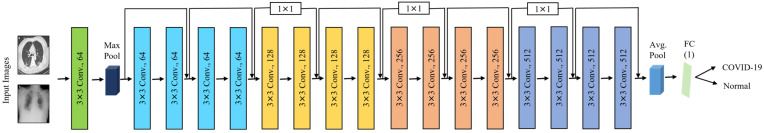
Network architecture of ResNet18 for COVID-19 detection.

ResNet18 model has been chosen for this study as it is the smallest residual neural network available, and it is also prominent in the medical image classification field [35]. The biggest advantage of using residual neural networks is that they do not have the problem of gradient vanishing even with the larger neural network. ResNet18 used in this study was pre-trained on the ImageNet database and can classify images into 1000 distinct categories, including cat, dog, car, and person. As a consequence, the model has already understood a wide variety of attributes from many images, and, therefore, it is suitable to be used for transfer learning. ResNet18 works efficiently due to its relatively shallow architecture for deep networks, allowing faster training without performance degradation. The key component of the ResNet18 structure is residual blocks, which use a shortcut to avoid the convolutional layer. Figure 6 presents the architecture of the network, where each residual block consists of two 3 × 3 convolutional layers, followed by the normalization layer and activation function. These two 3 × 3 convolutional layers can be skipped, and the input can directly connect with the final activation function [36]. Adam optimizer was used for the back backpropagation for adjusting the weights of all the neurons. This model can attain an excellent performance on the CT scan and CXR image classification as it uses residual block for faster processing, with batch normalization and identity connection to help the network with the vanishing point gradient problem [37]. ResNet18 model weights were fine-tuned separately on the CT scan and CXR datasets using transfer learning. For training, the weights were changed using the Adam optimization approach with a learning rate of 0.001 for training.

#### 3.3.5. Evaluation Criteria

In order to evaluate the performance of the models, they were evaluated on standard performance metrics, specifically, accuracy, precision, recall, and F1 score, presented in Equations (6)–(9), respectively. These measures are based on four important factors: True Positive (TP), True Negative (TN), False Positive (FP), and False Negative (FN). TP denotes the number of times the model accurately predicts the COVID-19 patient’s condition, while FP is the number of times the model incorrectly predicts that the healthy patient has COVID-19. FN is the number of times the model incorrectly predicts the COVID-19 patient as normal, and TN is the number of times the model correctly predicts the normal patient as normal. Furthermore, early stopping criteria were applied for deep learning models, which are discussed below.

Accuracy is the ratio of all correct predictions (COVID-19, Normal) to the total number of predictions; hence, it is given by

(6) $$\mathrm{Accuracy}=\frac{\mathrm{TP}+\mathrm{TN}}{\mathrm{TP}+\mathrm{TN}+\mathrm{FP}+\mathrm{FN}}$$

Precision is expressed as the ratio of accurately predicted COVID-19 patients to the total number of positive predictions.

(7) $$\mathrm{Precison}=\frac{\mathrm{TP}}{\mathrm{TP}+\mathrm{FP}}$$

Recall (or sensitivity) is the ratio of accurately predicted COVID-19 patients to total COVID-19 observations, denoted by

(8) $$\mathrm{Recall}=\frac{\mathrm{TP}}{\mathrm{TP}+\mathrm{FN}}$$

The F1 score is the weighted average of precision and recall, useful when class distribution is uneven. Therefore, the F1 score consists of both FP and FN, and it is defined as

(9) $$F1\;\mathrm{score}=2\times \frac{\mathrm{Precision}\;\times \;\mathrm{Recall}}{\mathrm{Precision}+\mathrm{Recall}\;}\;$$

## 4. Result and Discussion

In this work, two conventional machine learning and two deep learning methods were used to detect the COVID-19 disease using medical images. Table 1 and Table 2 show the performance of each method in CT scan and CXR images, respectively, in the format of (*μ* ± *σ*) where average classification values represented by *μ* and *σ* represent the standard deviation. By examining the results, it is seen that deep learning methods strongly surpassed the conventional machine learning models.

In the conventional machine learning methods, SVM with RBF kernel and cost (C) as 1 gives the best performance with an accuracy of 82% ± 1% and 77% ± 3% for CT scan and CXR images, respectively. The proposed SCovNet reached the best performance on all metrics, with the best accuracy of almost 99% for CT images and 98% for CXR images, which is extremely relevant.

**Figure 7:**
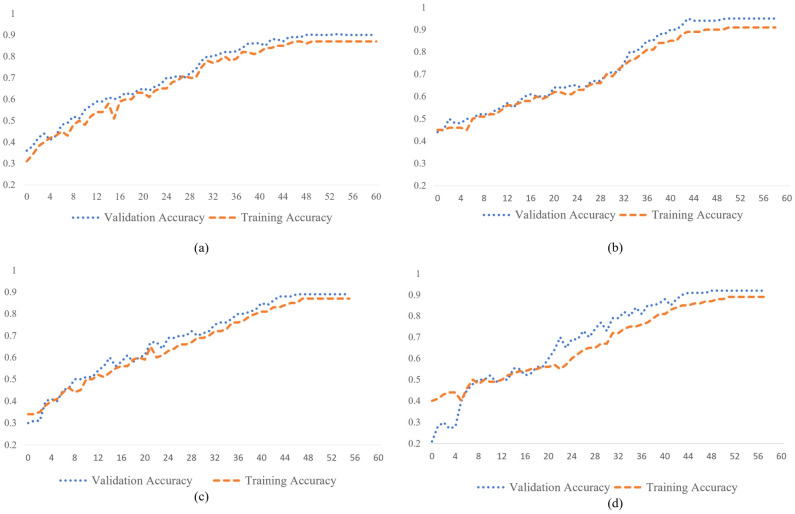
Training and validation curve of accuracy for the deep learning models on CT scan and CXR datasets: (**a**) SCovNet model for CT scan images; (**b**) ResNet18 model for CT scan images; (**c**) SCovNet model for CXR images; (**d**) ResNet18 model for CXR images.

Figure 7 shows the accuracy curve on training and validation sets against the batches processed for the deep learning models with both CT and CXR datasets. Early stopping (complete 10 continuous epoch cycles without change in the accuracy by 0.01) is applied in order to avoid the overfitting problem. In Figure 7, at the start, the training accuracy and validation accuracy are very low in both SCovNet and ResNet18. However, the accuracy curve of training and validation become similar at the end, indicating that both deep learning models are evolving positively during training, reaching the highest value of almost 92% and 96%, respectively, given by the proposed SCovNet for both CXR and CT scan images. Both deep learning models were run for a maximum of 60 epoch cycles and reached the optimal level of accuracy, as shown in Figure 7.

**Figure 8:**
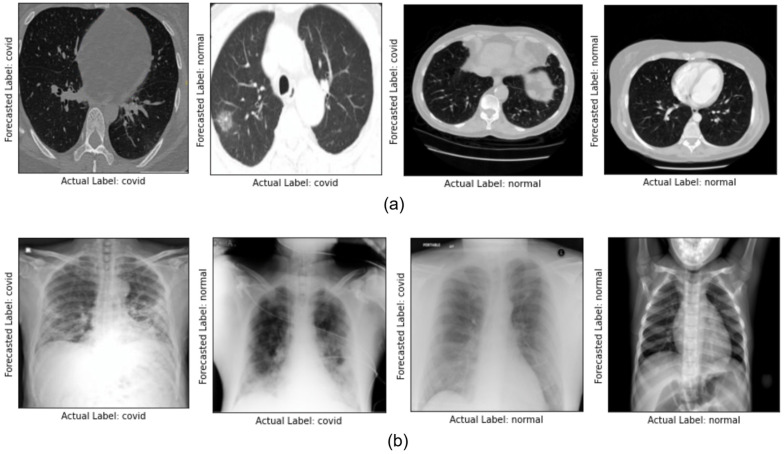
Prediction result of proposed SCovNet model (**a**) CT scan dataset and (**b**) CXR Images dataset.

The prediction of the proposed SCovNet model shows satisfying performance in classifying COVID-19 and normal patients for both datasets, but some of the images are miss-classified as some of them are low contrast images or in the presence of an artifact. Figure 8 shows the prediction result of the SCovNet model for all the possible scenarios in CT scan and CXR images. After reviewing the proposed SCovNet results, it is possible to conclude that the proposed architecture may be utilized as a replacement for the RT-PCR test, which is more time consuming in COVID-19 detection. Furthermore, ResNet18 was chosen in this study because it has almost the same architecture size as the proposed SCovNet, and it is also seen that the proposed SCovNet is smaller in size as compared to ResNet18 and still able to achieve the highest accuracy, which makes the proposed SCovNet model more likely to be used for COVID-19 detection for both the CT scan and CXR images.

**Figure 9:**
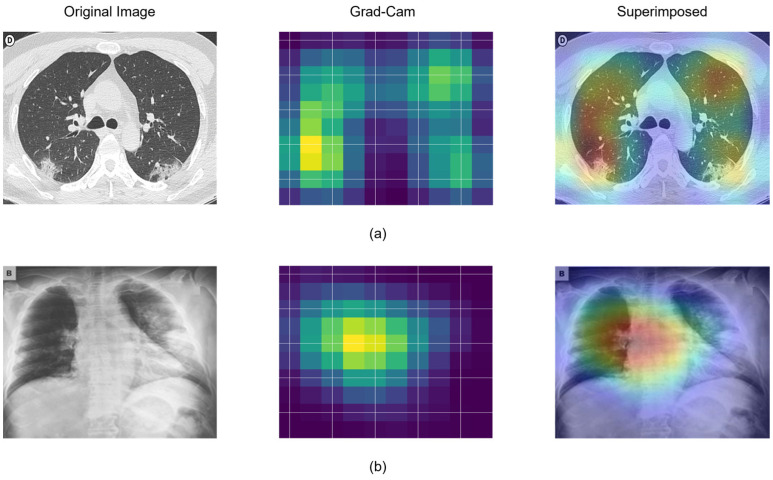
Grad-Cam visualization of the proposed SCovNet model for COVID-19 positive patient (**a**) CT scan and (**b**) CXR image.

Making the deep neural network more interpretive is also essential for many deep learning applications, particularly in the medical field. This study used Gradient-Weighted Class Activation Mapping (GradCAM) [38] to visualize the area of interest seen by the proposed SCovNet model in the CT scan and X-ray image, making it easier to understand for radiologists. The Grad-CAM approach gives a clearer picture of deep learning models. It may also be highly useful in COVID-19 detection because it reveals more about the model. Figure 9 highlights the region where the model concentrates its attention (region of interest) during the feature extraction process for both the CT scan and CXR images. After analyzing Figure 9, it is possible to say that the proposed SCovNet focused on the correct region of interest for the COVID-19 patient for both the CT scan and CXR.

This study is also compared with the recent works, shown in Table 3. Kassani et al. [21] and Kavitha et al. [24] combined pre-trained model with conventional machine learning for COVID-19 detection; they used a pre-trained model for feature extraction and conventional machine learning method for COVID-19 detection instead of only deep learning methods, achieving an accuracy of 99% and 99.15%, respectively. AI-Waisy et al. [20] achieved a significant accuracy of 99.99% using a hybrid deep learning framework COVID-CheXNet, which is basically a combination of two deep learning models ResNet34 and HRNet, but in their study, they used a dataset of a total 800 CXR images, which is smaller compared to our study. Panwar et al. [25] and Khan et al. [27] proposed novel frameworks called nCOVnet and CoroNet, respectively. They utilized enhanced pre-trained deep learning models by adding more convolutional layers for the classification. Alruwaili M et al. [22] improved the Inception-ResNetV2 framework and achieved an accuracy of 98.80% on CXR images (2905) dataset. Some of the previous studies show better results than the proposed work, but all of them worked on relatively smaller datasets compared to this study, which is very important for the generalized study.

It has also been discovered that CT scan pictures are more suited for COVID-19 identification since they provide more specific information about the patient’s health in CT scan; minor changes in the chest can also be observed, but on the other hand, CXR images are more often used in the medical field because they are easily available and cheaper in nature. However, this study shows that CT scan images are more reliable for COVID-19 detection.

## 5. Conclusions

As the number of COVID-19 pandemic patients grows by the day, with new variations entering the picture, it is critical to detect COVID-19 cases as soon as possible. In our proposed study, a machine and deep learning model, using CXR and CT scan images, is used to identify whether each patient is affected by COVID-19.

Our proposed work has been used to automatically identify COVID-19 and normal cases by using CXR and CT scan images based on the different machine and deep learning classifiers, namely, LR, SVM, SCovNet, and ResNet18. Out of all four classifiers, the proposed SCovNet architecture achieved a higher accuracy of 99% for CT scans and 98% for CXR images when compared with the other three models. Furthermore, after the generalization of our machine and deep models, deep learning models with CT scan images can help in the identification and prediction of COVID-19 diseases.

The significant outcomes were achieved from the proposed SCovNet model; however, this work could be improved by validating with other datasets. Our proposed model can also be useful for the identification of lung changes caused by COVID-19 infection. In addition to these, both CT scan and CXR could be combined for the same patient to achieve even better results. Additionally, future studies can be conducted to explore the impact of other laboratory tests and clinical features.

## Figures and Tables

**Table 1:** Overall performance assessment (*μ* ± *σ*) for CT scan images.

	Precision (%)	Recall (%)	Accuracy (%)	F1 Score (%)
Logistic Regression	72.23 ± 03.22	76.71 ± 02.11	73.10 ± 02.74	74.40 ± 02.49
SVM	77.13 ± 01.74	81.44 ± 02.39	82.38 ± 01.61	79.23 ± 02.09
ResNet18	94.14 ± 00.78	95.40 ± 01.51	98.20 ± 00.78	94.77 ± 01.02
**SCovNet**	96.34 ± 00.21	97.34 ± 00.47	98.67 ± 00.27	96.84 ± 00.32

**Table 2:** Overall performance assessment (*μ* ± *σ*) for CXR images.

	Precision (%)	Recall (%)	Accuracy (%)	F1 Score (%)
Logistic Regression	70.84 ± 02.48	75.12 ± 03.84	71.11 ± 03.03	72.92 ± 03.01
SVM	74.11 ± 02.54	78.47 ± 02.40	77.84 ± 03.03	76.23 ± 02.68
ResNet18	91.39 ± 01.48	93.24 ± 01.73	96.44 ± 00.42	92.31 ± 01.59
**SCovNet**	95.21 ± 00.74	96.74 ± 01.54	97.62 ± 00.20	95.97 ± 01.09

**Table 3:** Comparative study of proposed and previous work.

Related Work	Imaging Technique	Model	Dataset (Number of Images)	Accuracy
Jun Chen et al. [10]	CT scan	UNet++	46,096	96.00%
Jiao et al. [19]	CXR	EfficientNet	1834	84.60%
AI-Waisy et al. [20]	CXR	CheXNet (ResNet34+ HRNets)	800	99.99%
Kassani et al. [21]	CXR	DenseNet121 + Bagging tree classifier	274	99.00%
Alruwaili M et al. [22]	CXR	Enhanced Inception-ResNetV2	2905	98.80%
Hemdan et al. [23]	CXR	VGG19 and DenseNet201	50	90.00%
Kavitha et al. [24]	CT scan	ResNet-101 + LR	1545	99.15%
Panwar H et al. [25]	CXR	nCOVnet (VGG16 + CNN)	337	97.00%
Nguyen D et al. [26]	3D CT scan	CNN	-	98.80%
Khan A et al. [27]	CXR	CoroNet (Xception + CNN)	1251	89.60%
Bukhari S et al. [28]	CXR	ResNet-50	278	98.18%
Gouda W et al. [30]	CXR	CNN based on ResNet-50	21,165	99.63%
Misra S et al. [37]	CXR	Ensemble 3 ResNet-18	6008	93.90%
Proposed	CT scan	SCovNet	8055	98.67%
Proposed	CXR	SCovNet	9544	97.62%

## Data Availability

The dataset used in this article is openly available in Mendeley at https://data.mendeley.com/datasets/8h65ywd2jr.
